# Past, present, and future climate at select INDEPTH member Health and Demographic Surveillance Systems in Africa and Asia

**DOI:** 10.3402/gha.v5i0.19083

**Published:** 2012-11-23

**Authors:** David M. Hondula, Joacim Rocklöv, Osman A. Sankoh

**Affiliations:** 1 Department of Environmental Sciences, University of Virginia, Charlottesville, VA; 2 Department of Public Health and Clinical Medicine, Umeå Center for Global Health Research, Umeå University, Umeå, Sweden; 3 INDEPTH Network Secretariat, Accra, Ghana; 4 School of Public Health, University of the Witwatersrand, Johannesburg, South Africa; 5 Institute of Public Health, University of Heidelberg Medical School, Heidelberg, Germany

**Keywords:** INDEPTH, CLIMO, climate change, climatology, temperature, precipitation, seasonality, vulnerability, LMICs

## Abstract

**Background:**

Climate and weather affect human health directly and indirectly. There is a renewed interest in various aspects of environmental health as our understanding of ongoing climate change improves. In particular, today, the health effects in low- and middle-income countries (LMICs) are not well understood. Many computer models predict some of the biggest changes in places where people are equipped with minimal resources to combat the effects of a changing environment, particularly with regard to human health.

**Objective:**

This article documents the observed and projected climate profiles of select sites within the International Network for the Demographic Evaluation of Populations and Their Health (INDEPTH) network of Health and Demographic Surveillance System sites in Africa and Asia to support the integration of climate research with health practice and policy.

**Design:**

The climatology of four meteorological stations representative of a suite of INDEPTH Health and Demographic Surveillance Systems (HDSSs) was assessed using daily data of 10 years. Historical and future trends were analyzed using reanalysis products and global climate model projections.

**Results:**

The climate characteristics of the HDSS sites investigated suggest vulnerability to different environmental stressors, and the changes expected over the next century are far greater in magnitude than those observed at many of the INDEPTH member sites.

**Conclusions:**

The magnitude of potential future climate changes in the LMICs highlights the need for improvements in collaborative climate–health research in these countries. Climate data resources are available to support such research efforts. The INDEPTH studies presented in this supplement are the first attempt to assess and document associations of climatic factors with mortality at the HDSSs.

Ever since industrialization, the global climate has undergone rapid changes in patterns and distributions, and climate models predict further large-scale changes on the basis of greenhouse emission scenarios. Observations show that the global temperature has increased by 0.84°C over the past century (1) and the rate of rise in temperature is projected to increase over the coming decades (2). This global temperature trend is accompanied by a larger suite of climatic changes that will continue to impact people and the environment over the coming decades. The frequencies of extreme weather events, for example, are projected to increase in many regions of the world (3). Of increasing interest is determining the potential impacts of future climate change on human health and well-being. Weather and climate are key drivers of human mortality, morbidity, and migration in many locations across the globe, and as the global climate changes so will the impact on people, whether through direct mechanisms (e.g. heat-related deaths during heat waves) or indirect processes (e.g. long-term droughts restricting food supplies). Current knowledge of the relationship between climate and health varies geographically, and in some regions the linkages are poorly understood. These regions include low- and middle-income regions of Africa and Asia.

Residents of low- and middle-income countries (LMICs) are commonly identified as the most vulnerable to climate change because many of the projected changes are the most severe in regions where the people have limited resources for adaptation and mitigation (4, 5). Under this broad context, a growing literature identifies specific linkages between a wide range of climate factors and health outcomes in the developing world. As health surveillance programs continue to develop, reliable heath outcome data sets are becoming available and, as a result, researchers are able to link various diseases to climatic factors. One mature health surveillance network is the International Network for the Demographic Evaluation of Populations and Their Health (INDEPTH) in LMICs. This global alliance includes over 30 research centers in LMICs that host Health and Demographic Surveillance Systems (HDSSs) and provides continuous and regular monitoring of demographic variables, including birth rate, death rate, and migrations with the goal of ‘providing a better, empirical understanding of health and social issues, and to apply this understanding to alleviate the most severe health and social challenges’ (http://www.indepth-network.org).

Today, it is recognized that the health impacts related to climate variability and long-term change are severe, and that the health burden in the developing world could be lessened if these effects were more fully understood to promote strategic improvements to public health policy and practice. This calls for the integration of health science with climate and climate change science and associated partnerships amongst scientists from a variety of disciplines. In the developed world, where climate and health data are often more accessible, well-documented, and continuous over both space and time, this type of cross-disciplinary coordination of research efforts has led to public health infrastructural improvements that increase the capability of the population to recognize and mitigate primarily extreme events. Cities in developed countries across the globe have implemented heat health watch warning systems, for example, based on research demonstrating the positive relationship between human mortality and high temperatures during the warm season (6, 7). In LMICs, however, the picture is much different, as the integration of climate and health research has been inhibited by the availability of high-quality data from both the climate and health communities.

Our goal is to document the current climate profiles of select INDEPTH member sites that are participating in the Climate and Mortality (CLIMO) project featured throughout this supplement. The CLIMO collaboration offers researchers at specific INDEPTH member centers access to very high quality health and climate data to model current impacts of weather and climate on human health. In addition to assessing the present-day climatology, we also sought to investigate historical changes and projected future trends in several climate variables. In doing so, this manuscript aims to: (1) deepen the background on local climate to better understand the public health challenges related to climate and climate change and (2) document some of the climate data resources available to those studying health impacts in the LMICs.

## Methods

**Fig. 1:**
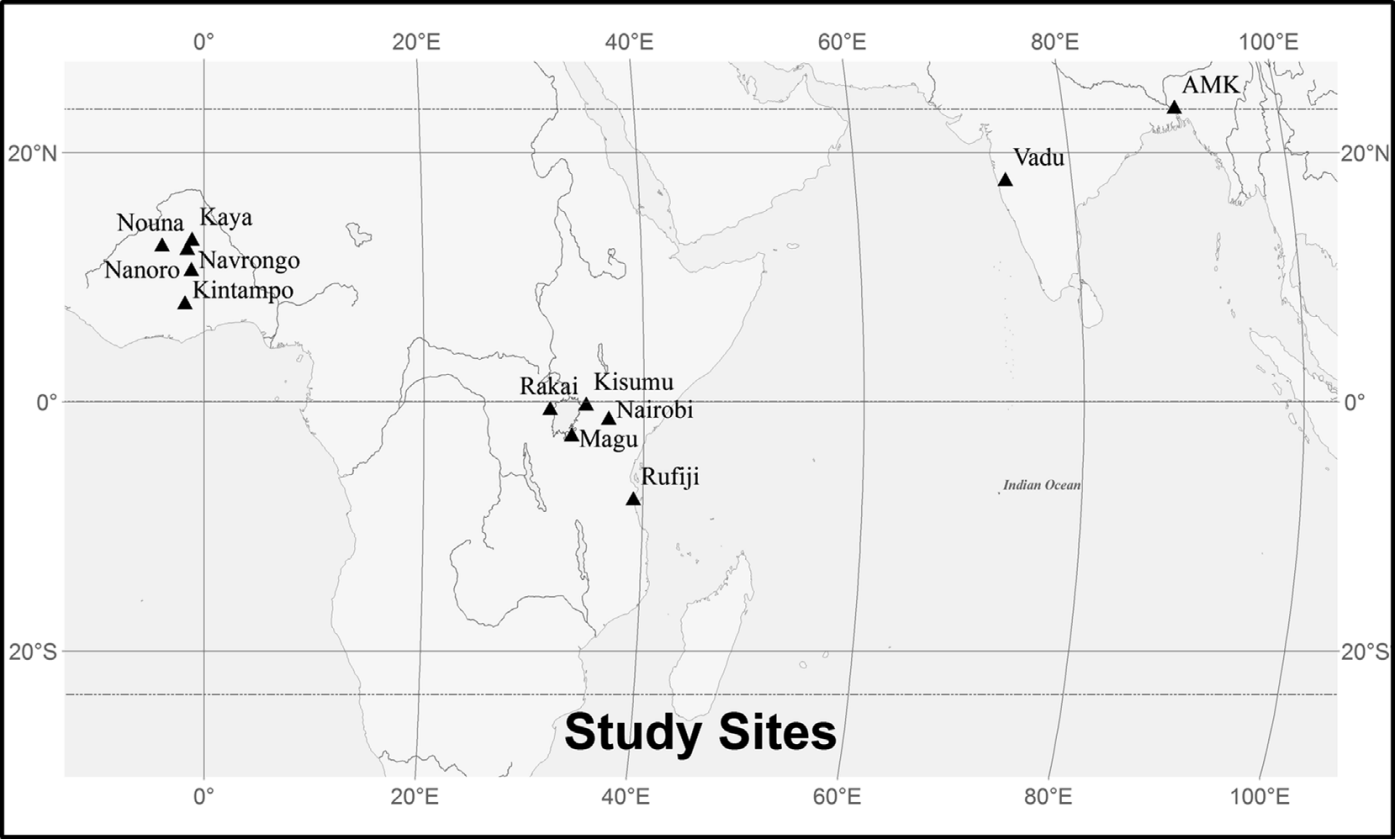
Locations of the 12 INDEPTH member HDSSs evaluated in this study.

The current climate of the INDEPTH member HDSS sites was evaluated via weather station data from four proximate sites that are representative of the regional climate of the HDSSs in the study (Table 1 and Fig. 1). We downloaded monthly station data from the US National Climatic Data Center's Global Historical Climatology Network, a publicly accessible compilation of quality-controlled weather station data from national and international agencies (www.ncdc.noaa.gov). Data from Agartala and Poona date back to 1901, but temperature measurements were reliably available only over the last 40 years of the record. Jomo and Boromo had shorter periods of record with the stations coming online in 1957 and 1945, respectively. Although the data sets are not 100% complete, beginning in the late 1970s there are very few cases where consecutive months are missing and it is rare that the same month is missing for multiple years within a decade. These stations represent the best available data at the monthly scale for evaluating the climate of these locations that we are aware of; the density of stations in these locations is relatively sparse. We examined eight variables: the number of days in each month with precipitation >0.25 cm (‘rain days’), the number of days in each month with >2.5 cm of precipitation (‘heavy rain days’), the total monthly precipitation, the mean monthly temperature, the mean monthly minimum and maximum temperature, and the monthly extreme minimum and maximum temperature.

**Table 1:** Location of INDEPTH member sites and proximate meteorological stations

Site	Country	Latitude	Longitude	Elevation (m)
INDEPTH HDSS and CLIMO sites				
*West Africa (Boromo meteorological station)*				
Kaya	Burkina Faso	13.08	−1.08	329
Nanoro	Burkina Faso	12.35	−1.52	295
Nouna	Burkina Faso	12.62	−3.82	276
Kintampo	Ghana	7.99	−1.72	352
Navrongo	Ghana	10.65	−1.15	198
				
*East Africa (Jomo Kenyatta meteorological station)*				
Kisumu	Kenya	−0.14	34.75	1188
Nairobi	Kenya	−1.28	36.82	1677
Magu	Tanzania	−2.62	33.46	1180
Rufiji	Tanzania	−7.75	39.17	70
Rakai	Uganda	−0.5	31.5	1278
				
*India (Poona meteorological station)*				
Vadu	India	17.89	73.92	91
				
*Bangladesh (Agartala meteorological station)*				
AMK	Bangladesh	23.7	90.42	11
				
Meteorological stations				
Agartala	India	23.883	91.25	16
Boromo	Burkina Faso	11.73	−2.92	264
Jomo Kenyatta	Kenya	−1.317	36.917	1624
Poona	India	18.533	73.85	559

Historical temperature trends at the sites were evaluated with reanalysis data from the Climatic Research Unit (CRU) downloaded from the Intergovernmental Panel on Climate Change (IPCC) Data Distribution Centre (http://www.ipcc-data.org). We extracted the decadal average mean annual temperature and precipitation for the 0.5°×0.5° grid cell that contained the latitude and longitude of each of the HDSS. These values were available from each of the 10 decades between 1901 and 2000. We similarly downloaded the decadal average yearly maximum and minimum temperature for the same time period. Trends were analyzed with linear regression; those trends with *p*-values = 0.05 were deemed significant.

A 30-year average temperature as well as precipitation projections was obtained from two different climate models and three future growth scenarios, also from the IPCC's Data Distribution Centre. We examined the UKMO HADCM3 and NCAR CCSM3 model output under the A1B, A2, and B1 scenarios. Annual average air temperature and precipitation values were available along with monthly averages by decade for each 30-year interval. We were not able to obtain 30-year maximum or minimum temperatures for this particular combination of models and scenarios. Projections were merged with historical data by scaling the projection for the first decade to the most recent observation from the 1990s. In cases where the historical data exhibited a significant trend, the projection was based on the last historical observation plus or minus a continuation of the linear trend until the time point of the first projection.

We also evaluated the current and future climate using the Köppen Climate Classification. Köppen is the most commonly utilized global classification scheme and the system was updated in 2006 to be more compatible with current reanalysis data sets and projections (8). The system, originally developed in 1900, relates the climate to vegetation and identifies five major climate types: equatorial, arid, warm temperate, snow, and polar. Within each of these broad categories, there are additional classification characteristics based on temperature and precipitation, resulting in a total of 31 different climate regimes that cover the entire globe. Climate classification systems like Köppen provide a different perspective for evaluating climate shifts by providing a more integrated view of multiple variables simultaneously. A change in classification type is indicative of substantial changes in a suite of climate variables that would impact vegetation types, and more broadly impact the environmental systems of the area with implications for human health and welfare. Applying future climate models to the Köppen classification system indicates some degree of change across all latitudes and climate types, with approximately 3% of the current polar climate shifting to a warmer snow climate by 2100 and 1.7% of the current warm temperate climate in the lower latitudes shifting to an arid climate related to a reduction in precipitation (using the A1F1 and B1 scenarios) (9). We downloaded the global GIS shapefiles of Köppen types for the years 2000–2025 and 2076–2100 using A1F1, A2, B1, and B2 scenarios run with the Tyndall Centre for Climate Change Research model from http://koeppen-geiger.vu-wien.ac.at/shifts.htm. We then extracted the classification type for the grid cell containing the coordinates of each of the INDEPTH member sites.

## Results

### Current climate

All 12 INDEPTH member sites we investigated fall within the tropics and subtropics; the station farthest from the equator Abhoynagar, Mirsarai, and Kamlapu (AMK in Bangladesh) falls just north of the Tropic of Cancer at 23.7°N. Thus, all of the sites experience high temperatures throughout the year, with very few days near or below freezing across the entire network. Residents of the INDEPTH study areas are regularly exposed to high temperatures, but future changes in the distribution of high temperatures could directly impact human health via additional thermal stress and indirectly through modifying the rate and likelihood of disease transmission throughout the population. Precipitation variability across the sites was found to be greater than temperature variability because of the relative positioning of the sites to major water bodies and predominant air flows.

**Fig. 2:**
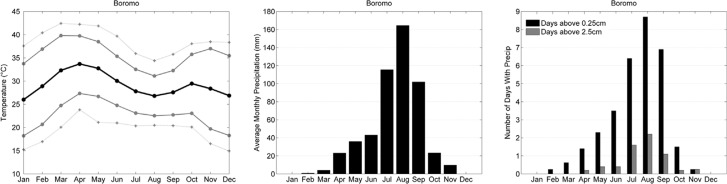
Climate characteristics of Boromo, Burkina Faso, 2000–2009. (a) shows the temperature seasonality. The solid black line represents the mean monthly temperature. The upper and lower solid gray lines represent the mean monthly maximum and minimum temperature, respectively. The dashed gray lines represent the extreme monthly maximum and minimum temperature. (b) shows the precipitation seasonality with the average monthly precipitation (mm). (c) shows the precipitation seasonality based on the number of days with rainfall greater than 0.25 cm (black bars) and 2.5 cm (gray bars) per month.

The western Africa INDEPTH member sites (Kaya, Nouna, Nanoro, Navrongo, and Kintampo) were represented through analysis of the Boromo weather station in Burkina Faso (see Fig. 1). This region experiences a seasonal pattern, where the temperature peaks in both April and October. The highest mean, maximum, and extreme temperatures are observed during these months, with a tendency for spring to be slightly warmer than the fall (Fig. 2).

The summer months at these locations are characterized by high precipitation totals with 3 months of >100 mm each and lower temperatures as cloud cover suppresses incoming solar radiation and evaporation of ground and atmospheric moisture contribute to cooling. This strong seasonality is driven by the intertropical convergence zone (ITCZ), a latitudinal band of low pressure and convective precipitation that circles the globe and migrates northward and southward across the tropics following the seasonal progression of the sun. During the summer, the ITCZ moves northward bringing regular convective activity and precipitation to the West African INDEPTH sites. In the winter months, the northeasterly trades become more persistent as the ITCZ moves southward and dry air is advected from interior northern Africa. Between November and March, these sites experience <1 day of 0.25 cm of precipitation per year (Fig. 2).

**Fig. 3:**
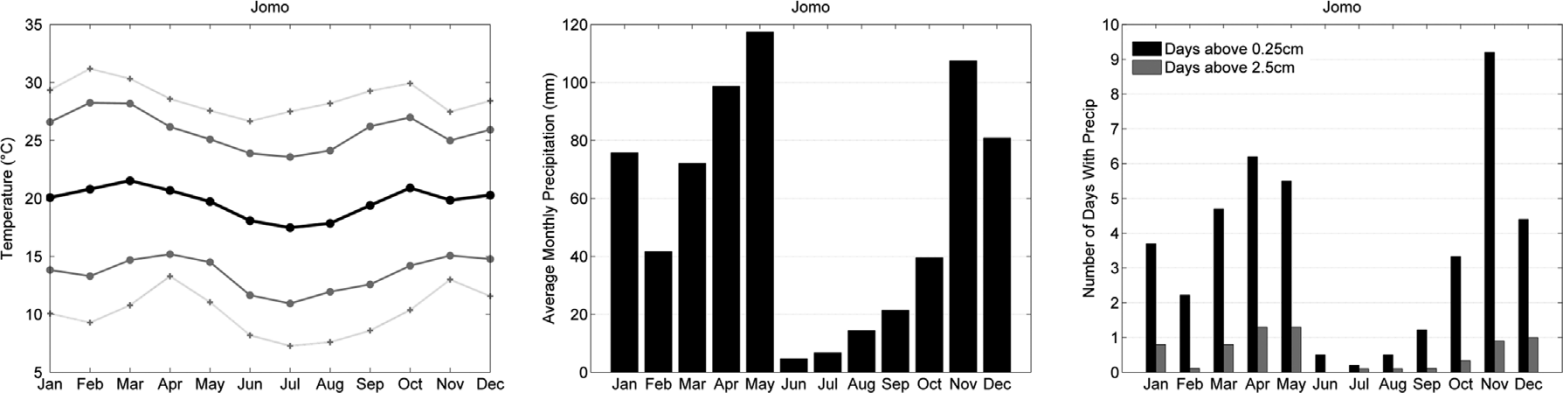
Climate characteristics of Nairobi, Kenya, 2000–2009 as in Fig. 2.

The climate at the East African INDEPTH member sites (Rakai, Kisumu, Nairobi, Magu, and Rufiji) was evaluated with meteorological data from Jomo Kenyatta airport in Nairobi. The data from this airport are typical of an equatorial climate with the mean monthly, minimum, and maximum temperatures very consistent throughout the year (Fig. 3). The temperature is lower than would otherwise be expected for equatorial Africa because of the high elevation: Jomo Kenyatta is located at approximately 1,500 m above sea level and the three closest sites are roughly 1,000 m above sea level. The Rufiji site, the farthest south of this cluster, is located less than 100 m above sea level and thus the data from Jomo Kenyatta are less representative of Rufiji's coastal climate.

The precipitation pattern for these stations is contrary to that of the West African sites. Here the majority of the precipitation falls from November through May, with very few precipitation days in the months of June, July, and August. The months with the most precipitation are May and November, but November experiences more days with significant rainfall (>0.25 cm) on average than May. The double precipitation maximum arises from the ITCZ passing through the region twice a year. Average annual precipitation at these sites approaches 700 mm.

**Fig. 4:**
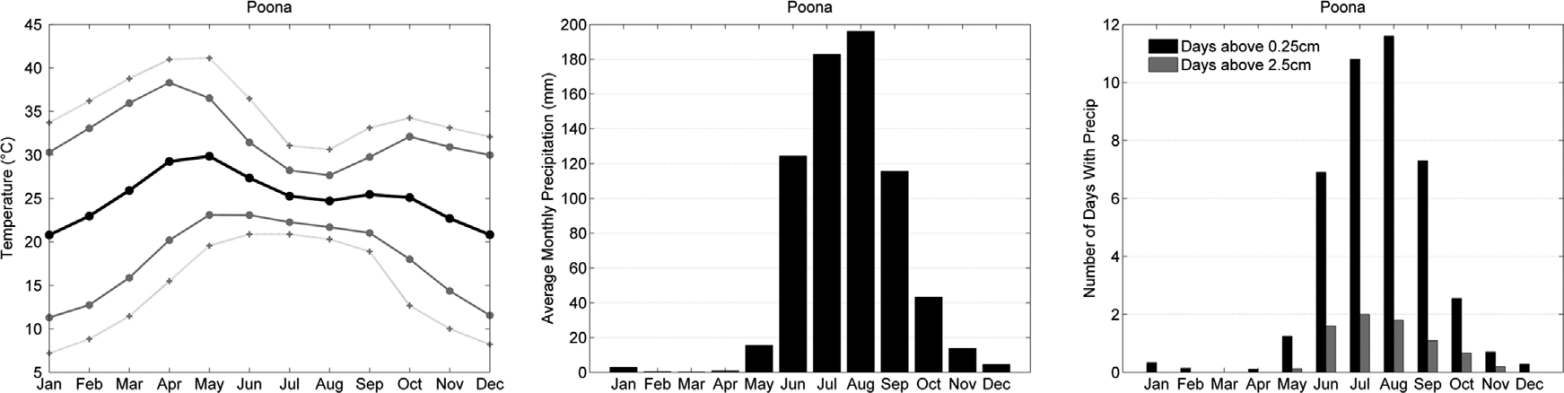
Climate characteristics of Vadu. India, 2000–2009 as in Fig. 2.

The climate of the Vadu site was evaluated with data from the nearby Poona meteorological station and is similar in temperature and precipitation characteristics to the West African sites (Fig. 4). Temperatures are relatively high throughout the year with monthly maximum temperatures >30°C and extremes in the vicinity of 40°C in the spring. Mean monthly temperatures are highest in the spring months.

Like much of the Indian subcontinent, Poona's precipitation pattern is driven by the regional monsoon. As the land surface over the Asian continent warms in the summer months and the ITCZ moves northward, low pressure persists in the region and moist air is advected northward from the Indian Ocean. As a coastal site, Poona has direct access to summer moisture, with 4 months averaging >100 mm each. The most significant and heavy precipitation days fall in the wettest months of June to September. The regional flow completely reverses in the winter months and accordingly there is nearly no rainfall between December and April.

**Fig. 5:**
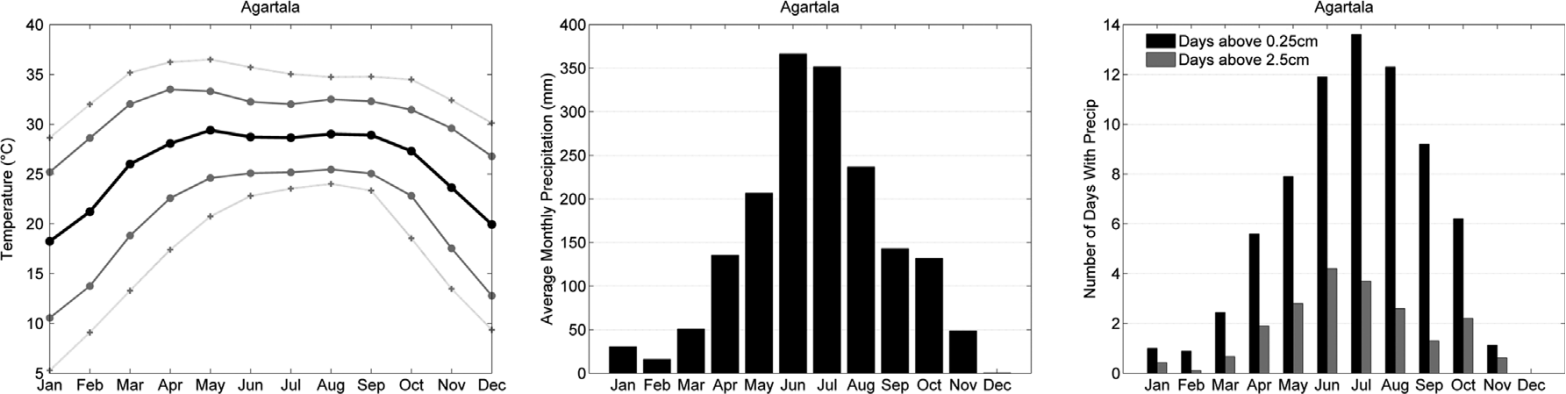
Climate characteristics of Agartala, India, 2000–2009 as in Fig. 2.

Data from the Agartala meteorological station in northeastern India were used to represent the climate of the AMK site in Bangladesh (Fig. 5). Temperature seasonality at Agartala is more characteristic of the northern hemisphere subtropics, with the highest temperatures in the months of May through September and a decline to minimum temperatures in December and January. During the warm season, temperatures may be quite high, with monthly maxima >30°C and extremes upwards of 35°C. Temperatures are somewhat moderated from other locations in the subtropics because of Agartala's proximity to the coast.

The Agartala/AMK region experiences some of the highest monthly precipitation totals observed anywhere on the planet, with >350 mm falling on average in the months of June and July. As with Poona, the thermal low over the Asian continent and the northward position of the ITCZ drive moist air into the region, but here the site is located much closer to the Himalayan mountains, which enhance uplift and dramatically increase precipitation totals. This south Asian summer monsoon is one of the most significant climatic phenomena with respect to human health and well-being, as the region boasts some of the highest population densities on the planet that are impacted by the extreme precipitation and regular flooding (10).

### Historical and future changes

Change in climate at the INDEPTH member sites could have important implications in terms of public health, and, as such, we used observational data and projections to examine past and expected future changes. With respect to the historical temperature record, the decadal annual mean temperature time series only identified significant increases at 2 of the 12 study sites, AMK and Vadu. At 8 of the 12 sites, however, the 1990–2000 decadal average temperature was the highest of the time series. The lack of significant trends at some of the sites might be attributed to the coarse time increment, small sample size, and other decadal-scale variability. Using decadal average temperatures, we cannot conclude that significant warming has occurred at most of the INDEPTH HDSS sites over the 20th century. At the majority of sites, the total range in decadal average temperatures was <1°C.

**Fig. 6:**
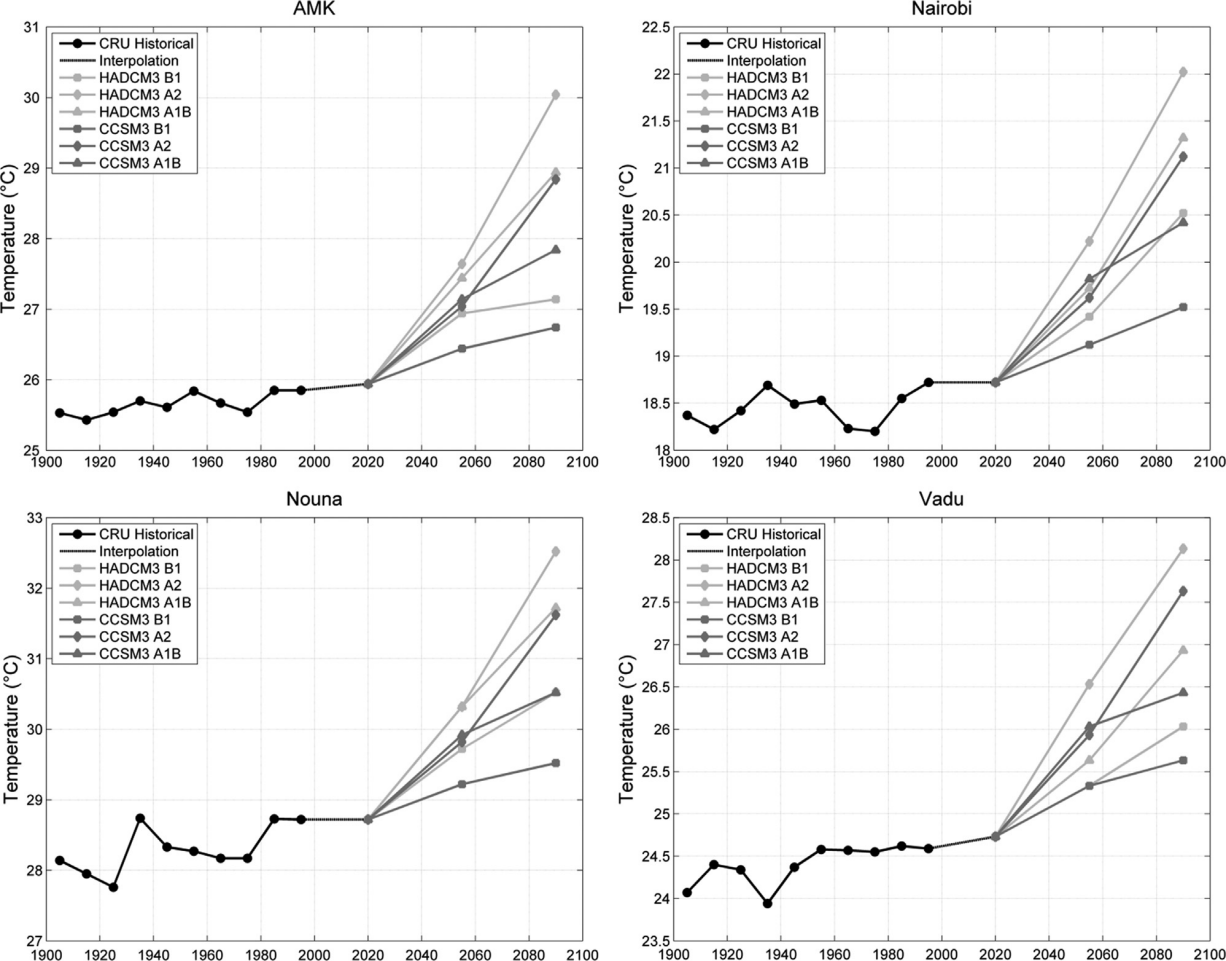
Historical and projected future temperatures at four INDEPTH member sites. The black dots represent the mean decadal temperature for each decade of the 1900s based on the Climate Research Unit reanalysis record. The dashed line extrapolates the historical data to the start of the modeling period; a slope in the dashed line indicates a statistically significant linear trend from 1900–2000. The gray lines each show mean temperature projections for twenty-year intervals from the HADCM3 and CCSM3 models for three different climate change scenarios. All points are shown at the midpoint of the appropriate time interval.

Future projections for all sites, however, place year 2100 temperatures above the 1990–2000 decadal average across all six model–scenario combinations evaluated (Fig. 6). Even more striking is that the rate of temperature change is projected to increase dramatically. In many cases, the temperature increase over the next century is expected to be 2–3°C. The most conservative model–scenario combination (CCSM3 A1B) predicts increases of 0.5–1°C. The highest projections were typically associated with the HADCM3 model and the A2 scenario.

**Fig. 7:**
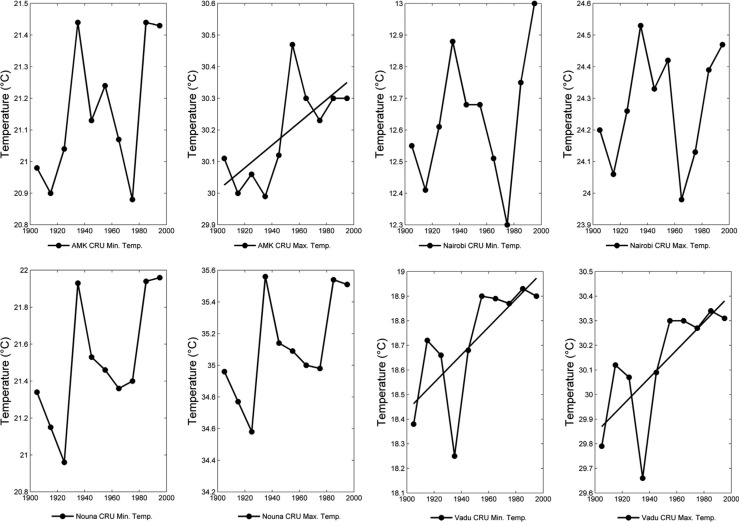
Historical mean decadal minimum (left) and maximum (right) temperatures at four INDEPTH member sites, 1900–2000, based on CRU reanalysis data. A line is drawn for cases where simple linear regression indicated a statistically significant trend in the historical data. All points are shown at the midpoint of each decade.

We also investigated historical trends in decadal average maximum and minimum annual temperature. As with mean temperature, only 2 of the 12 stations (Vadu and AMK) showed a statistically significant trend using simple linear regression through the 10 data points, and we caution the robustness of such results given the small sample size. As with mean temperature, we found that at the majority of the stations, the highest minimum and maximum temperatures were found in the most recent decade of the time series (Fig. 7).

**Fig. 8:**
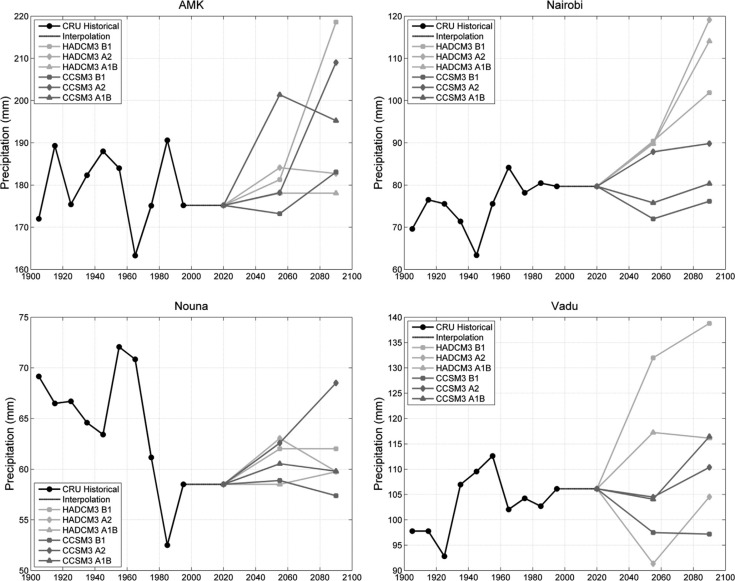
Historical and projected future precipitation (mm per month) at four INDEPTH member sites, similar to Fig. 6.

No significant trends were evident in the decadal-scale time series of annual average precipitation, with the exception of Kintampo where we found a significant decline. There was no tendency for the 1990–2000 decadal average precipitation to fall above or below the rest of the time series across the network of 12 stations (Fig. 8).

Projections for future annual precipitation averages varied considerably across models and scenarios (Fig. 8). At most stations, there was some disagreement on the *sign* of the expected change between model–scenario combinations, with even further discord on the magnitude of the changes. At Nairobi, all three HADCM3 projections indicate an increase in precipitation over the next century, with year 2100 annual averages falling between 20 and 50% above the current average. Changes of this magnitude would have severe consequences for the regional water budget. Only one of the CCSM3 model runs (A2) is consistent with the HADCM3 projections, while the A1B and B1 scenarios show a slight decrease in precipitation through mid-century and then a return to present-day averages by year 2100. At AMK and Nouna, the majority of the projections indicate an increase in precipitation over the next century, while projections for Vadu were nearly evenly split amongst increasing, no change, and decreasing precipitation.

**Fig. 9:**
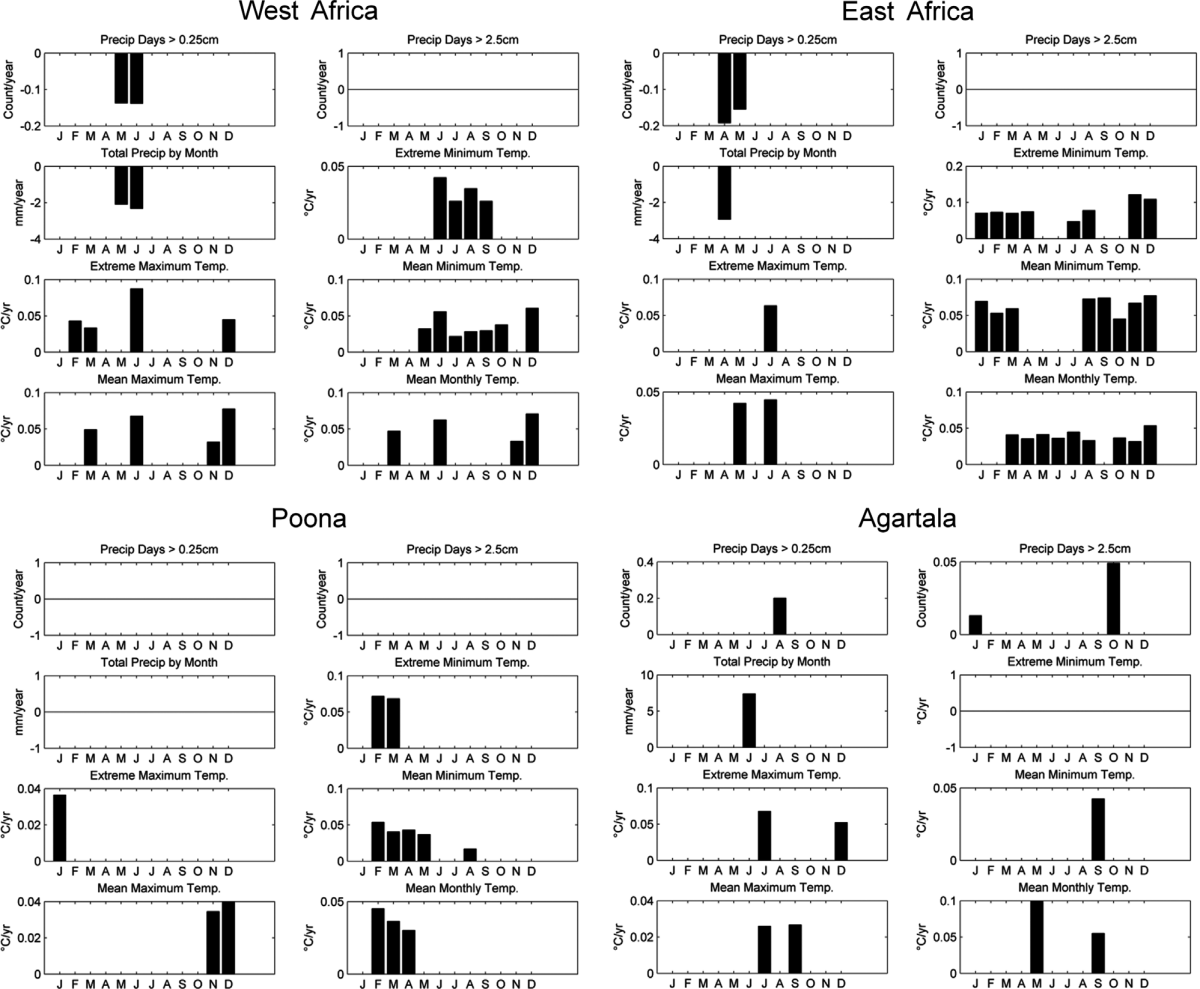
Trends in historical climate characteristics by month at meteorological stations proximate to INDEPTH member sites. A bar is drawn in all cases where simple linear regression through the yearly time series data (1979–2011) indicated a significant trend: the bar height corresponds to the regression slope B. For each station, the panels show trends per month in (a) the number of days with precipitation >0.25 cm, (b) the number of days with precipitation above 2.5cm, (c) total precipitation, (d) extreme minimum temperature, (e) extreme maximum temperature, (f) mean minimum temperature, (g) mean maximum temperature, and (h) mean monthly temperature.

We returned to the GHCN-Monthly station data to determine if there were significant trends in any of the eight variables we evaluated for the current climate profiles of the INDEPTH member sites over recent decades (Fig. 9). At the West African sites, there was limited evidence of changes in the seasonality of precipitation, with significant negative trends for the months of May and June. These are the months immediately preceding the wet season, and, thus, although they do not account for a significant portion of the total annual precipitation, they do represent rain at the end of the dry season that provides some of the first relief. The magnitude of the trends (approximately −2 mm/year) is substantial compared to the approximately 40 mm/month of rainfall received at this station during May and June. Changes in the temperature distribution point toward higher minimum and extreme minimum temperatures during the warm and wet seasons. Mean and maximum temperatures have increased during certain months in the cool season and higher means and maximums are also evident in June.

Historical trends in precipitation at Jomo Kenyatta are limited to April and May, with April showing a decrease in rain days and total monthly precipitation. There is evidence of changes in the temperature distribution throughout the year, with either higher extreme or mean minimum temperatures observed in all months except May and June. Mean monthly temperatures have increased throughout most of the year as well. In general, there are no significant trends in mean or extreme maximum temperatures at this site.

We did not find evidence of any changes in precipitation days or total at Poona when the analysis was divided by month. Minimum and mean temperatures were found to be increasing in the late winter and early spring months, with higher maximum temperatures in the mid-winter. There were very few significant trends present for Agartala.

Using the Köppen climate classification, we identified major shifts at 2–3 of the 12 sites investigated using the A1F1, A2, B1, and B2 scenarios in the Tyndall temperature and precipitation projections for the 21st century. Under the ‘A’ scenarios, two sites are associated with a change in climate classification, Nairobi and Vadu. The shift in Nairobi is from the ‘Cfb’ type (Warm temperate fully humid [no dry months], warm summers) to ‘Aw’ (Equatorial with dry winter). The change in type is suggestive of higher temperatures throughout the year and a decrease in wintertime moisture. At Vadu, the change is also to 'Aw' but here the shift is from 'Am', Equatorial monsoon, indiciative of a possible increase in moisture in the winter months. Under the ‘B’ scenario, Rakai was added as a site with a projected change, moving from Am to Aw. We found that the number of sites with changes was sensitive to the start value used: the 1951–2000 historical average differed from the four model projections for the 2001–2025 time period in a few cases. Furthermore, within the model projections for the first quarter of the 21st century, there were differences between the four scenarios.

**Fig. 10:**
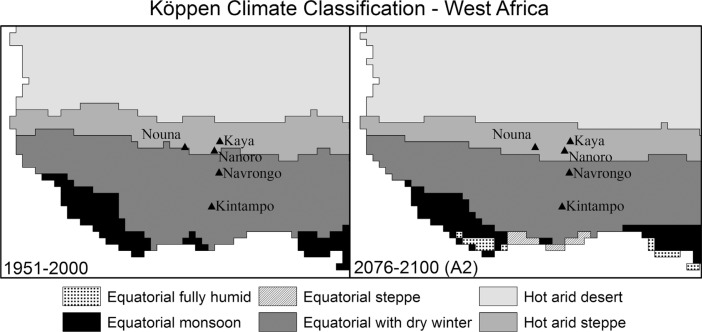
Estimated Köppen climate classification types in West Africa for (a) the second half of the twentieth century and (b) the final 25 years of the 21st century projected with the Tyndall climate model and the A2 scenario.

A site that does not experience a change in Köppen types may still undergo a change in climate that impacts human health. The classification scheme is discrete, whereas changes are likely to occur over a continuous gradient. Under the A2 scenario, for example, none of the five West African INDEPTH member HDSSs show a change in the climate regime. But, examination of the map indicates that the regional climate is clearly varying – the zone of hot arid steppe climate, for example, migrates southward over the study period and the hot arid desert zone's southern edge encroaches on the study region (Fig. 10). Farther south, away from the INDEPTH sites, the equatorially fully humid climate regime appears in the years 2076–2100, with no regional presence in the past half-century.

## Discussion

The climates of the INDEPTH member HDSSs we examined are relatively diverse given their similar latitude. This is consistent with previous work that demonstrated that the African INDEPTH HDSSs represent a wide range of environmental and climatic regimes across the continent (11). All of the sites we examined are representative of warm climates, as expected for tropical and subtropical locations, but the strong seasonality in temperature and precipitation add an additional dimension of vulnerability to weather and climate. Cold weather effects, for example, might occur at Jomo Kenyatta, Poona, or Agartala, where the temperature may fall into the single digits during a few months of the year. The sensitivity of the populations near Agartala and Boromo to high temperatures may differ because Boromo has a consistently warm climate throughout the year (so there are no ‘unusual’ heat events), whereas the temperature at Agartala is much more seasonally variable.

With respect to precipitation, at the West African sites, >75% of the total annual precipitation falls in the three months of July, August, and September. Locations like these with a highly seasonal climate could be most sensitive to a change in the seasonality of rainfall or a disruption in precipitation patterns during the rainy season. Continued improvement of seasonal projections in precipitation may be especially helpful in preparing these locations for climate change, as changes in the seasonal climate pattern can have devastating effects on agricultural production and human health (12). The health of people at the West African sites might be more impacted by a decrease in precipitation relative to those in eastern India and Bangladesh, where rainfall is more abundant. Like the West African sites, these locations demonstrate considerable seasonality in precipitation. In contrast, however, populations in eastern India and Bangladesh may be more sensitive to increases in precipitation, as flooding events are, as such, regular occurrences.

Changes to Earth's climate over the past and coming century will manifest in many different ways across the globe. Although the general trend is toward a warmer planet, the specific rate of warming can vary considerably, and in some locations there have even been a slight cooling trend (13). Thus, the result we are presenting that all of the HDSSs have not seen statistically significant warming using decadal-scale temperature data should not be entirely surprising. The gridded global temperature trends shown in the Fourth Assessment Report of the Intergovernmental Panel on Climate Change (IPCC FAR, 14) show the greatest changes over the northern hemisphere high latitudes, with only slight trends over many of the regions where the HDSSs we examined are located. Further, in some of these regions, the IPCC FAR acknowledges that there are no sufficient data to produce reliable trends. In terms of projections of the future climate, similar spatial heterogeneity exists. The African continent as a whole is expected to warm, in many places at a faster rate than the global mean. The same is true for southern Asia. Considerable uncertainty exists regarding future precipitation in the regions where INDEPTH member sites we examined are located (14).

LMICs are commonly identified as being especially sensitive to climate change, and many of the model projections for the INDEPTH member sites indicate that the rate of temperature change will accelerate considerably over the coming decades. From a health perspective, significant impacts from climate change might occur via a large range of different mechanisms (15). An increase in temperature will directly add thermal stress, for example, and if the change is manifested through increased and more severe extreme heat events, the population may not have time to acclimatize to new extremes. If the change is manifested through a more consistent increase throughout the year, the spread of diseases and food and water resources may be impacted as the change in temperature impacts the hydrologic cycle.

The potential health impacts of climate change are not only dependent on changes in the means and extremes but also on changes to the timing of high and low temperatures and precipitation events. Adding rainfall in the wet season for the Vadu and AMK sites, for example, would have significantly different (and negative) consequences relative to adding moisture during the half of the year when nearly none falls. We sought to use the CRU, HADCM3, and CCSM3 datasets to evaluate changes in seasonality using a similar framework as above but found that the modeled current and future seasonality patterns were too different from observations (and across models/scenarios) for useful analysis. The magnitude of the differences between models and scenarios was far greater than any projected change in seasonality, and in many cases the warmest or wettest months of the year were not correctly depicted by the modeled data. Using regional instead of global climate models to evaluate changes in seasonality is recommended for future studies examining such possible changes in these locations. This particular analysis suggests that resources be devoted toward infrastructural adaptations that accommodate precipitation variability in either direction until model certainty improves.

It is our intention that this climatological survey of the CLIMO sites serves as a useful resource for those who focus on climate–health issues throughout the INDEPTH network and other populations in LMICs. Future researchers may find the data sources we used – weather station data obtained from the US National Climatic Data Center's online portal and reanalysis and model projection data from the IPCC data distribution center – beneficial in their own work. Both resources are freely available and present differing strengths and challenges. The weather station data are true measurements obtained from point locations, and in many cases are available at various time scales (hourly, daily, or monthly) even in LMICs. However, at some stations, particularly in the regions we examined, the records often contain long periods of missing values. The reanalysis data from the IPCC data distribution center can be seen as complementary to the station observations. Working with these derived gridded data not only provides continuous spatial and temporal coverage but also adds uncertainty regarding the representativeness of grid cell values for individual locations. The same scaling uncertainty is present in the gridded model projections as well, with an added layer of uncertainty related to making a prediction for the future. Our recommendation is that station data be used wherever possible, but in regions where meteorological observing sites are sparse, other validated products may still be useful in exploring climate change or climate–health linkages.

We acknowledge that while we have provided several analyses to highlight elements of the past, current, and future climates at these locations, each analysis in this manuscript could be improved upon in some way. Most notably, the data we used are readily available but are at coarse spatial and temporal scales, and this limited the application of statistical techniques to identify variability and trends. Downscaling coarsely measured trends to specific locations is an imperfect process with high levels of uncertainty, and, thus, the changes we have presented in this manuscript should not be viewed as tailored predictions for the individual INDEPTH HDSSs, but instead as examples of the types of changes that could occur in these regions. The IPCC FAR explicitly acknowledges considerable uncertainty in downscaled projections for these regions (14). Nonetheless, these projections can and should be examined in synthesis with research examining climate–health linkages, including those presented in this supplement, to gain a sense of perspective regarding potential climate-driven changes in population health. Policymakers and planners can use this information to anticipate the range of future health burdens and develop strategies to minimize the impact of environmental changes on human health. Research groups with expertise in regional climate models could make a useful contribution in preparing and reporting expected changes in the seasonality and precipitation at these locations, including assessments of the uncertainty, based on models with a higher spatial and temporal resolution, and indeed much ongoing effort is devoted to this very challenge (16). The long-term historical trends could also be reanalyzed using daily data (where it is available) which, in turn, could be merged with model projections on a daily scale. Furthermore, while we documented inter-site variability, there is likely also intra-site variability, as the HDSS are regions spanning hundreds of square kilometers. Intra-site variability in microclimates could impact vulnerability and could be assessed via remote sensing resources or improved meteorological monitoring networks. The Rufiji and Kintampo sites are located a considerable distance from the meteorological station they were linked to, and, thus, the results are likely to be least representative of those two HDSSs. Our focus was to provide an easily accessible guide to the climate at these sites. We also strongly encourage a deeper level of study of both the climate, and perhaps more importantly, climate–health linkages in these locations.

## Conclusion

Climate data resources are available to study the relationship between weather and health across the 12 HDSS sites participating in the INDEPTH CLIMO initiative. Although very few sites exhibited significant trends in temperature or precipitation using decadal-scale data from the past 100 years, climate models predict large changes in both variables at many locations. Projections for all sites consistently pointed toward a warm climate over the next century, but there was little agreement between climate models and scenarios in how precipitation might change in these locations. Across all sites, projections indicate that the climate may change dramatically in the coming years relative to the past century. Some sites may experience changes of a magnitude large enough to change their climate classification type. Collaborative efforts to link climate and health data sets to understand the sensitivity of low- and middle-income populations to various climate and weather phenomena can help guide adaptation efforts for those who might be most vulnerable as the global climate changes.
